# Short-Term Starvation Weakens the Efficacy of Cell Cycle Specific Chemotherapy Drugs through G1 Arrest

**DOI:** 10.3390/ijms24032498

**Published:** 2023-01-28

**Authors:** Munan Shi, Jiajia Hou, Shan Shao, Weichu Liang, Shiwei Wang, Yuzhou Yang, Zhigang Guo, Feiyan Pan

**Affiliations:** Jiangsu Key Laboratory for Molecular and Medical Biotechnology, College of Life Sciences, Nanjing Normal University, 1 Wen Yuan Road, Nanjing 210023, China

**Keywords:** short-term starvation, etoposide, GAPDH, cell cycle

## Abstract

Short-term starvation (STS) during chemotherapy can block the nutrient supply to tumors and make tumor cells much more sensitive to chemotherapeutic drugs than normal cells. However, because of the diversity of starvation methods and the heterogeneity of tumors, this method’s specific effects and mechanisms for chemotherapy are still poorly understood. In this study, we used HeLa cells as a model for short-term starvation and etoposide (ETO) combined treatment, and we also mimicked the short-term starvation effect by knocking down the glycolytic enzyme GAPDH to explore the exact molecular mechanism. In addition, our study demonstrated that short-term starvation protects cancer cells against the chemotherapeutic agent ETO by reducing DNA damage and apoptosis due to the STS-induced cell cycle G1 phase block and S phase reduction, thereby diminishing the effect of ETO. Furthermore, these results suggest that starvation therapy in combination with cell cycle-specific chemotherapeutic agents must be carefully considered.

## 1. Introduction

Malignant tumors destroy the normal physiological functions of tissues and organs and seriously threaten human health and life [1,2]. Since the vulnerability of cancer cells to nutritional deficiencies and their dependence on specific metabolites have become new features of cancer, starvation therapy provides a new idea for malignancy treatment by starving cancer cells by blocking their nutritional supply to the tumor [3,4]. The fasting/fasting-mimicking diet (FMD) is characterized by half the calories, low glucose and protein, and high fat content, which can sensitize cancer cells to different treatments while protecting normal cells from toxic agents [5,6,7]. Moreover, starvation therapy enhances efficacy by synergizing with oxidative therapy, chemotherapy, sonodynamic therapy, and inhibition of tumor cell autophagy [8,9,10,11,12].

The etoposide (VP-16) targets DNA topoisomerase II activities by stabilizing a covalent topoisomerase II-cleaved DNA intermediate complex (Top2cc) in the catalytic cycle of the enzyme, leading to DNA breaks and cell death [13,14,15]. In addition, proteolysis of the trapped Top2cc is a key step to expose and repair these lesions [16,17,18,19,20]. Etoposide is widely used in treating small-cell lung cancer and testicular cancer, but the development of resistance to etoposide is also a major problem in clinical treatment [21,22]. The MDM2-SNP-mediated downregulation of Topo II limits the drug sensitivity of ETO [23]. In addition, fasting protects multiple small intestinal stem cell populations marked by *Lgr5*, *Bmi1*, or *HopX* expression and maintains barrier function to preserve small intestinal architecture from the high dose of etoposide [24].

The glyceraldehyde-3-phosphate dehydrogenase (GAPDH), one of the housekeeping genes, catalyzes the conversion of glyceraldehyde 3-phosphate to D-glycerate 1,3-bisphosphate in glycolysis [25]. In addition to this long-established metabolic function, GAPDH has recently been implicated in several non-metabolic processes, including transcription activation, apoptosis, membrane fusion, and DNA repair [26,27,28]. In comparison with normal tissues, tumor cells rely more on aerobic glycolysis to produce more ATP to maintain their high proliferation rate, known as the Warburg effect [29,30]. However, the mechanistic effects of GAPDH on glucose utilization remain unclear. Further, under glucose starvation, GAPDH is methylated at R234 by CARM1 to inhibit glycolysis and the proliferation of liver cancer cell lines [31]. Meanwhile, AMPK-dependent phosphorylation of GAPDH is essential for glucose starvation-stimulated Sirt1 activation and autophagy [32]. The AKR1B10 negatively regulates autophagy by reducing GAPDH upon glucose starvation in colon cancer [33]. The above GAPDH is sensitive to changes in intracellular glucose content; whether it responds to FMD and the exact mechanism are obscure.

In this study, we demonstrated that fasting did not enhance the efficacy of the chemotherapeutic agent ETO but rather reduced ETO-induced DNA damage and weakened the therapeutic effects of ETO. A fasting-mimicking diet significantly downregulated GAPDH expression, and the knockdown of GAPDH similarly reduced ETO-induced DNA damage and attenuated the effects of ETO, suggesting that the reduction of GAPDH could partially mimic the effect of FMD. We further found that short-term starvation or GAPDH knockdown reduced the effect of ETO by inducing cell cycle G1 phase block and S phase reduction. Our study not only provides a theoretical explanation for the effects of starvation therapy and related mechanisms but also suggests that starvation therapy needs to be carefully evaluated in combination with different chemotherapeutic agents in clinical treatment.

## 2. Results

### 2.1. Short-Term Starvation Protects Cancer Cells against Chemotherapy Drug ETO

**Figure 1:**
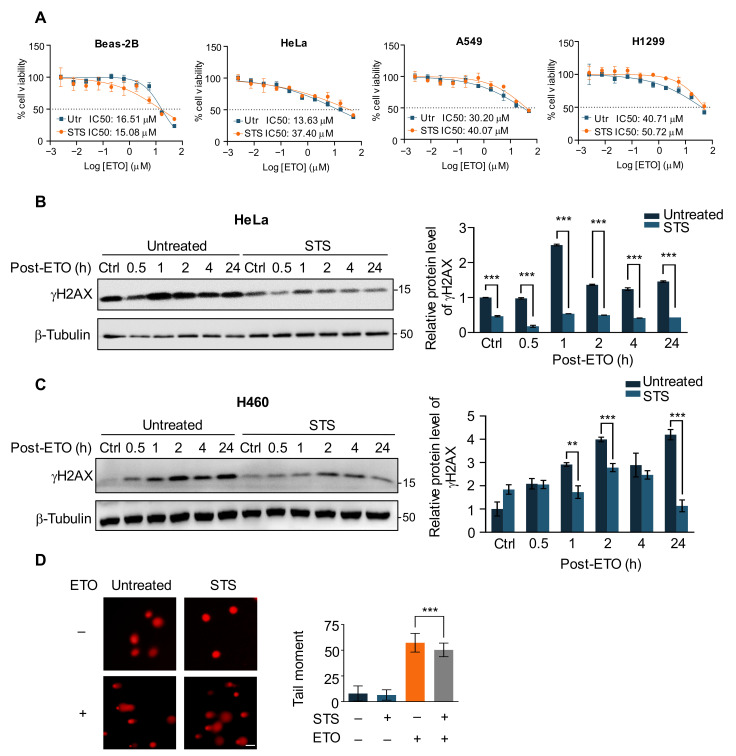
Short-term starvation protects cancer cells against ETO by reducing DNA damage. (**A**) Dose-response curves with IC50 values that indicate cell lines pre-starved or left untreated for 24 h were treated with the indicated concentration of ETO for 48 h. (**B**) Immunoblot (**left**) and quantification (**right**) of γH2AX in HeLa cells pre-starved or untreated for 24 h with 20 μM ETO treatment for 2 h following different recovery times. (**C**) Immunoblot (**left**) and quantification (**right**) of γH2AX in H460 cells pre-starved or untreated for 24 h with 20 μM ETO treatment for 2 h following different recovery times. (**D**) Comet assay images (**left**) showing the tail moment (**right**) of pre-starved or untreated HeLa cells with or without 20 μM ETO treatment for 2 h. Scale bar, 100 μm. Data is represented as mean ± SD of at least three independent experiments. *p* values are from student’s *t*-tests. ** *p* < 0.01; *** *p* < 0.001.

In order to investigate the role of fasting in the response of cancer cells to chemotherapy drug treatment, we first used the short-term starvation (STS) medium of 0.5 g/L glucose and 1% FBS in DMEM to pre-starve HeLa cells and two human lung cancer cell lines, A549 and H1299, for 24 h, versus normal Beas-2B cells treated with different concentrations of the chemotherapy agent etoposide (ETO) for 48 h [6]. The results showed that the IC50 value of the cancer cells in the starvation group was significantly higher than that of the control cells in the presence of increasing concentrations of ETO (Figure 1A). Similar results were also shown for the cell cycle-specific topoisomerase I inhibitor camptothecin, but the opposite held with the cell cycle-independent chemotherapeutic drug adriamycin (Supplementary Figure S1A,B). ETO is a semisynthetic derivative of podophyllotoxin, which inhibits DNA synthesis via topoisomerase II inhibition activity [34]. Therefore, to explore the mechanism of starvation to alleviate the role of cancer cells in ETO-induced apoptosis, we pre-starved Hela cells for 24 h after adding 20 μM ETO treatment for 2 h and collected cell lysates at different recovery time points of 0.5 h, 1 h, 2 h, 4 h, and 24 h, respectively. The histone H2AX is rapidly phosphorylated at the Ser139 site of DNA damage by PI3K-like kinases when DNA damage occurs; hence, γH2AX is commonly used as a marker for DNA double-strand breaks (DSBs) [35]. The results demonstrated that ETO treatment consistently increased γH2AX levels in HeLa and H460 cells, causing persistent DNA damage to cancer cells; however, under STS, γH2AX levels in the starvation group were lower than those in the control at all time points, indicating that short-term starvation protected cancer cells against ETO-induced DNA damage (Figure 1B,C). The comet assay can effectively detect and quantify the extent of a DNA break at the individual cell level and is widely used as an accepted method for DNA damage detection [36]. Similarly, starvation-treated cells produced fewer DNA breaks (Figure 1D), suggesting that short-term starvation protects cancer cells through the reduction of DNA damage caused by ETO.

### 2.2. GAPDH Is Involved in the Protection of Cells from ETO-Induced Cell Apoptosis by STS

**Figure 2:**
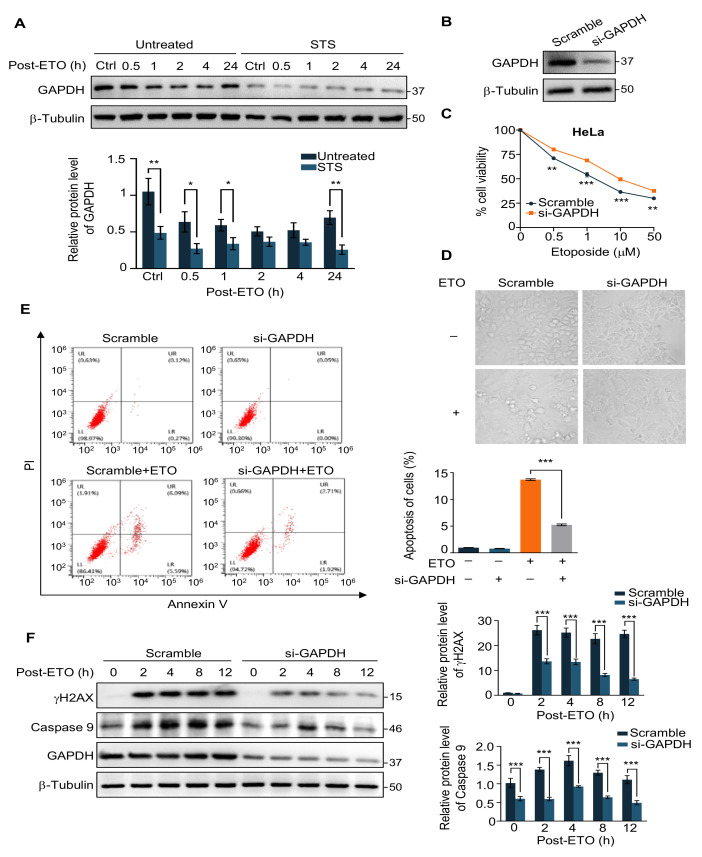
Knockdown of GAPDH mimics the protective effect of STS in response to ETO. (**A**) Immunoblot (**upper**) and quantification (**lower**) of GAPDH in HeLa cells pre-starved or untreated for 24 h with 20 μM ETO treatment for 2 h following different recovery times. (**B**) Immunoblot of GAPDH in HeLa cells transfected with scramble siRNA and si-GAPDH for 48 h. (**C**) Cell viability of HeLa cells transfected with scramble siRNA and si-GAPDH for 24 h was treated with the indicated concentration of ETO for 24 h. (**D**) Morphological analysis of control or GAPDH knockdown HeLa cells with or without ETO treatments (20 μM, 2 h). (**E**) Annexin V/PI staining and flow cytometry assay (**left**) and quantification (**right**) of control or GAPDH knockdown HeLa cells with or without ETO treatments (20 μM, 2 h). (**F**) Immunoblot (**left**) and quantification (**right**) of indicated proteins in control or GAPDH knockdown HeLa cells with 20 μM ETO treatment for different recovery times. Data represented as mean ± SD of at least three independent experiments. *p* values are from student’s *t*-tests. * *p* < 0.05; ** *p* < 0.01; *** *p* < 0.001.

Starvation decreases glycolysis and inhibits the catalytic activity of GAPDH, a key enzyme in glycolysis [31]. Consequently, we detected GAPDH protein levels in cells before and after starvation treatment in the presence of ETO, and a significant decrease was observed in the starvation group (Figure 2A). Next, we knocked down intracellular GAPDH with small interfering RNA (siRNA) to explore whether reduced glycolysis could mimic the protective effect of STS on ETO-induced apoptosis (Figure 2B). The cell viability assays verified that knockdown of GAPDH increases sensitivity to ETO (Figure 2C). A morphological analysis further validated these results, which showed that GAPDH deficiency made the cells crumple and diminished adherence (Figure 2D).

Apoptosis, also known as “programmed cell death,” is an orderly cell death that occurs under genetic regulation to maintain the stability of the internal environment [37]. Anti-tumor drugs such as ETO can trigger apoptosis in cancer cells [38]. We applied flow cytometry to detect apoptosis in GAPDH-knockdown HeLa cells and found that the control cells showed a significant increase in apoptosis under ETO treatment, while knockdown of GAPDH alleviated ETO-induced apoptosis (Figure 2E). The western blot analysis of the apoptosis marker Caspase 9 yielded the same results (Figure 2F). The above suggests that the downregulation of GAPDH expression is consistent with the effect of starvation on the cellular response to ETO, which can partially mimic the effect of short-term starvation.

### 2.3. GAPDH Knockdown Decreases ETO-Induced DNA Damage

**Figure 3:**
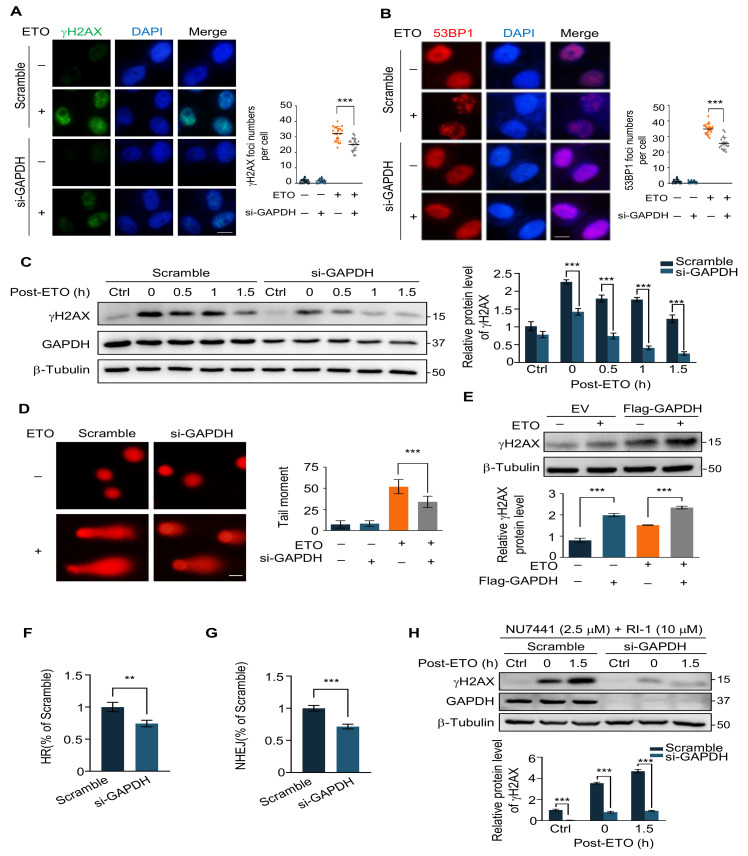
GAPDH deficiency protects cancer cells is not attributed to increased DNA damage repair. (**A**) Immunofluorescence images (**left**) of γH2AX in HeLa cells transfected with scramble siRNA or si-GAPDH after 20 μM ETO treatment for 2 h. DNA was stained with DAPI (blue). Quantification (**right**) of γH2AX foci/cell. Scale bar, 10 μm. (**B**) Immunofluorescence images (**left**) of 53BP1 in HeLa cells transfected with scramble siRNA or si-GAPDH after 20 μM ETO treatment for 2 h. DNA was stained with DAPI (blue). Quantification (**right**) of 53BP1 foci/cell. Scale bar, 10 μm. (**C**) Immunoblot (**left**) and quantification (**right**) of γH2AX in control or GAPDH knockdown HeLa cells with 20 μM ETO treatment for different recovery times. (**D**) Comet assay images (**left**) showing the tail moment (**right**) of control and GAPDH knockdown HeLa cells with or without 20 μM ETO treatment. Scale bar, 100 μm. (**E**) Immunoblot (**upper**) and quantification (**lower**) of indicated proteins in HeLa cells transfected with an empty vector and Flag-GAPDH were treated with or without 20 μM ETO. (**F**) The relative HR efficiency of U2OS cells were transfected with scramble siRNA and siGAPDH. (**G**) The relative NHEJ efficiency of U2OS cells was transfected with scramble siRNA and siGAPDH. (**H**) Immunoblot (**upper**) and quantitative analysis (**lower**) of γH2AX levels in HeLa cells transfected with scrambled siRNA and si-GAPDH for 24 h were treated with NU7441 and RI-1 for 2 h, and then treated with ETO following different recovery times. Data represented as mean ± SD of at least three independent experiments. *p* values are from student’s *t*-tests. ** *p* < 0.01; *** *p* < 0.001.

We have experimentally demonstrated that the knockdown of GAPDH enhances the survival of cancer cells in response to ETO and attenuates ETO-induced apoptosis. In addition, we next examined the cellular DNA damage in the control and GAPDH-knockdown groups. We transfected HeLa cells with scramble siRNA and si-GAPDH, and 48 h after transfection, the cells were treated with ETO (20 μM, 2 h), followed by immunofluorescence experiments to photograph the foci formation of γH2AX under confocal microscopy. Knockdown of GAPDH significantly reduced the number of γH2AX foci compared to the scramble (Figure 3A). Subsequently, we also examined the formation of 53BP1 foci, another marker of DSB, and, consistent with γH2AX, the number of 53BP1 foci was markedly decreased in the GAPDH knockdown cells (Figure 3B). This result indicates that knockdown of GAPDH reduced ETO-induced DNA double-strand breaks. We further determined the intracellular γH2AX protein levels by Western blot. HeLa cells were treated with ETO (20 μM, 2 h) after transfection with GAPDH siRNA for 48 h and then replaced with normal medium to continue the culture, and the samples were collected at the time points of 0 h, 0.5 h, 1 h, and 1.5 h of recovery, respectively. The results revealed that the γH2AX levels were significantly lower in the knockdown GAPDH group than in the control at all time points of recovery (Figure 3C). Meanwhile, the comet assay has reaffirmed the above results in both the scramble and GAPDH knockdown groups (Figure 3D). Moreover, to clarify the role of GAPDH in the cellular response to ETO-induced DNA damage, we adopted an overexpression strategy to detect changes in γH2AX induced by ETO in cells before and after GAPDH overexpression. The western blot revealed that overexpression of GAPDH increased the protein level of ETO-induced γH2AX (Figure 3E). In summary, the intracellular GAPDH concentration influenced the level of ETO-induced DNA damage.

The findings described above led us to hypothesize whether the increase in DSB repair efficiency caused this reduction effect. There are two main pathways to repair the DSB damage in mammalian cells: one is homologous recombination repair (HRR) and the other is non-homologous end joining (NHEJ) [39]. In order to measure the efficiency of DSB repair, we applied the HR repair reporter system (DR-GFP) as well as the NHEJ reporter system (EJ5-GFP) established based on U2OS cells [40], GAPDH siRNA was transfected in U2OS reporter cells for 24 h followed by transfection with I-SceI plasmid, and the ratio of GFP-positive cells was determined by flow cytometry after 24 h of continued incubation. It can be seen that GAPDH knockdown reduced the efficiency of HR and NHEJ (Figure 3F,G), which rules out the possibility of diminished total DNA damage due to increased efficiency of intracellular DSB repair.

Additionally, since the reduction in intracellular DNA damage is not caused by enhanced DNA damage repair, we asked whether the initial DNA damage induced by ETO treatment differs in the control and GAPDH knockdown groups. We selected specific inhibitors of NHEJ and HR, NU7441 (a DNA-PKcs inhibitor) and RI-1 (a RAD51 inhibitor), to pretreat the two groups of cells 2 h in advance and then changed to ETO for 2 h. Notably, γH2AX was lower in the GAPDH knockdown group compared to the scramble, indicating that there was less DNA damage within the GAPDH knockdown group when both DSB repair pathways were blocked and that ETO had a weaker effect on inducing DNA damage in the absence of GAPDH (Figure 3H). Moreover, we ruled out that GAPDH deficiency caused etoposide to induce more Top2cc that were not recognized or sufficiently removed from DNA to be converted into DNA double-strand breaks and therefore showed no elevated DNA damage markers or effects of HR or NHEJ inhibitors (Supplementary Figure S2A) [41,42].

### 2.4. G1 Phase Arrest Weakens the Efficacy of Chemotherapy Drugs

**Figure 4:**
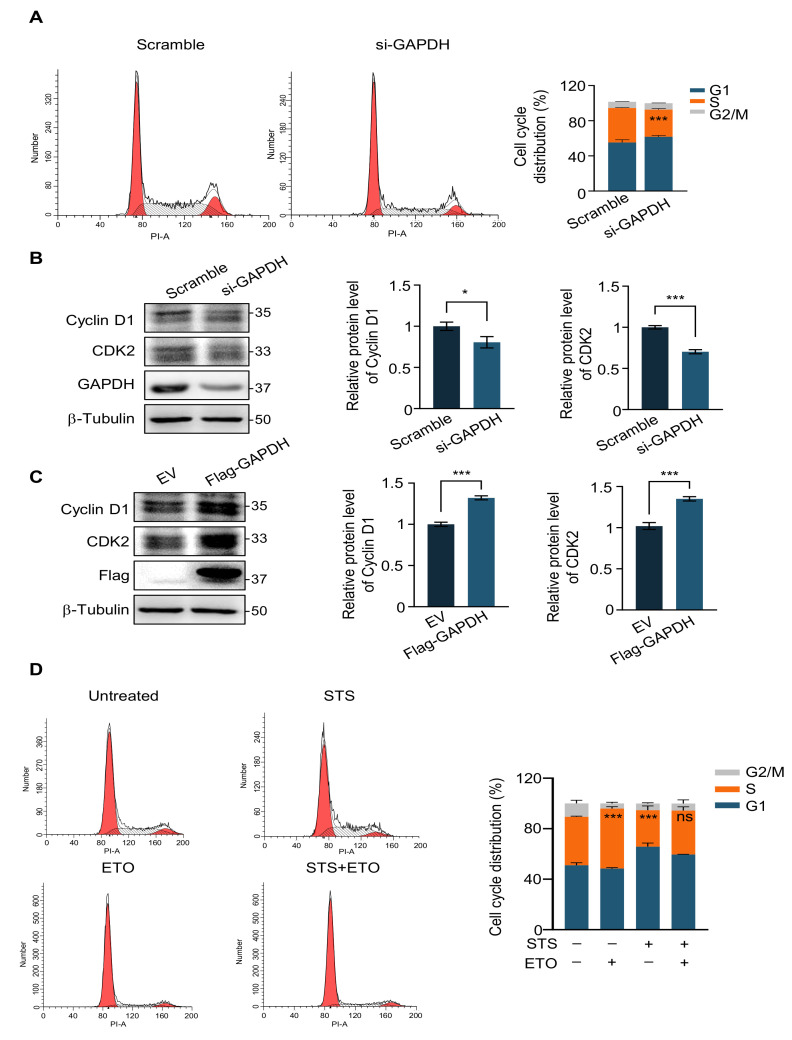
G1 phase arrest diminishes the efficacy of chemotherapeutic agents. (**A**) Cell cycle analysis (**left**) and quantification (**right**) of HeLa cells transfected with scramble siRNA or si-GAPDH. (**B**) Immunoblot (**left**) and quantification (**right**) of indicated proteins in HeLa cells transfected with scrambled siRNA and si-GAPDH for 48 h. (**C**) Immunoblot (**left**) and quantification (**right**) of indicated proteins in HeLa cells transfected with an empty vector and Flag-GAPDH for 48 h. (**D**) Cell cycle analysis (**left**) and quantification (**right**) of HeLa cells pre-starved or untreated for 24 h with or without 20 μM ETO treatment for 2 h. Data represented as mean ± SD of at least three independent experiments. *p* values are from student’s *t*-tests. * *p* < 0.05; *** *p* < 0.001 and ns: not significant.

While impeding DNA damage repair, etoposide induces S-phase accumulation and G2/M arrest, eventually resulting in apoptosis through the p53-related pathway in the mouse fetal brain [43]. We speculated that the effect of GAPDH knockdown on weakening ETO may have affected the cell cycle. In order to test our hypothesis, we transfected HeLa cells with scramble siRNA and GAPDH siRNA for 48 h and then applied flow cytometry to detect the cell cycle. Compared to the scramble, GAPDH knockdown resulted in an increased ratio of G1 phase cells and a decrease in the S phase, indicating that GAPDH deficiency caused G1 phase arrest (Figure 4A). In addition, we used Western blot to detect the expression of cell cycle proteins Cyclin D1 and CDK2. The results showed that GAPDH knockdown significantly down-regulated the expression of both, indicating that the cell transition from the G1 to the S phase was reduced (Figure 4B). We also performed GAPDH overexpression experiments to fully illustrate its cell cycle regulation. We found that when GAPDH was overexpressed, the expression of Cyclin D1 and CDK2 was correspondingly upregulated, demonstrating that GAPDH promoted the cells to enter the S phase from the G1 phase (Figure 4C).

Given that GAPDH affects ETO efficiency by influencing the cell cycle, we explored whether STS could also influence the effect of ETO through cell cycle blockade. The cell cycle distributions of the ETO treatment group alone reduced the G1 phase and increased the S phase of the cells only marginally, while the starvation treatment group alone and the starvation combination ETO treatment group significantly increased the G1 phase and reduced the S phase of the starved cells (Figure 4D and Figure S1C–F). 

## 3. Discussion

Malignant tumors are characterized by uncontrolled cell proliferation, infiltration, and aggressiveness, which seriously damage the normal physiological functions of tissues and organs and threaten human health and life [44,45]. Traditional treatments include surgery, chemotherapy, and radiotherapy, each of which has its limitations in clinical practice and needs to be complemented by other therapeutic methods in combination with synergistic treatment [46,47]. The Warburg effect, in which tumor cells prefer glycolysis for metabolism under aerobic or anaerobic conditions, consumes large amounts of glucose without efficiently producing energy [48,49]. In addition, given the high dependence of tumor cells on glucose, protein inhibitors of the glycolytic pathway are increasingly being developed [50]. Further, fasting inhibits the malignant progression of colorectal cancer by impairing aerobic glycolysis [51]. Our study found that in ETO-treated HeLa cells, starvation did not increase sensitivity to ETO but rather increased the survival of cancer cells under ETO treatment. The immunofluorescence results for the DNA damage maker proteins γH2AX and 53BP1 showed that short-term starvation reduced ETO-induced DNA double-strand break damage, which was further confirmed by alkaline comet assays.

Given the significant role of GAPDH in glucose starvation, to investigate the molecular mechanism of the protective effect of short-term starvation on HeLa cells, we first analyzed the expression of the GAPDH protein under STS treatment. Next, we knocked down GAPDH to explore whether it could partially mimic the effects of starvation. In addition, consistent with the predicted results, the absence of GAPDH enhanced the survival of HeLa cells under ETO treatment, and both ETO-induced apoptosis and DNA damage were significantly attenuated. GAPDH has been reported to be involved in DNA damage repair, for which we examined the repair efficiency of DNA double-strand breaks and found that GAPDH knockdown significantly impaired the repair efficiency of HR and NHEJ. Furthermore, after blocking both HR and NHEJ repair pathways, we found that the abbreviation of DNA damage after GAPDH knockdown was not caused by an enhancement of DSB repair efficiency but originated from the reduction of DNA damage caused by ETO induction.

The ETO is a cell cycle-specific drug that acts on DNA topoisomerase II to form a stable, reversible drug-enzyme-DNA complex that impedes DNA repair. In order to investigate the reason why GAPDH knockdown reduced ETO-induced DNA damage, we first found that GAPDH knockdown caused a reduction in the S phase and a block in the G1 phase. This finding was also confirmed by the decrease in Cyclin D1 and CDK2, suggesting that GAPDH knockdown induced cell cycle arrest and reduced S-phase, thus weakening the effect of ETO. Similarly, we found that STS induced cellular G1 phase block and S phase reduction, which provides a mechanistic explanation for its attenuation of the effect of chemotherapeutic agents. Given that there are multiple enzymes involved in the glucose metabolism process, it is unclear in this paper whether short-term starvation acts exclusively through GAPDH. In addition, the specific mechanism by which starvation downregulates GAPDH still needs to be further explored.

In summary, we explored the efficacy of short-term starvation against the chemotherapeutic agent etoposide ETO using cervical cancer cells HeLa as a model. We made a preliminary exploration of the mechanisms involved and found that STS protects cancer cells against the chemotherapeutic agent ETO by reducing DNA damage, which is associated with a decrease in GAPDH levels and cell cycle arrest. Since ETO is widely used in clinical practice [13,52,53], it is suggested that starvation therapy’s reported positive synergistic effects with conventional chemotherapy are not applicable to all cancers, and starvation therapy remains controversial. Therefore, combining starvation therapy with different chemotherapeutic agents in clinical treatment still needs to be evaluated carefully.

## 4. Materials and Methods

### 4.1. Cell Culture and Regent

The human cervical cancer cell line HeLa cells, the human lung cancer cell lines A549, H1299, and H460, and the human normal bronchial epithelium cell line BEAS-2B were purchased from ATCC. The HeLa, U2OS, and BEAS-2B cells were cultured in DMEM medium (Gibco, Cat#11965092, Carlsbad, CA, USA) supplemented with 10% FBS (Biochannel, Cat#BC-SE-FBS01, Nanjing, China) and 1% penicillin/streptomycin. In addition, the A549, H1299, and H460 were grown in RPMI-1640 medium (Gibco, Cat#11875119, Carlsbad, CA, USA) supplemented with 10% FBS and 1% penicillin/streptomycin (Solarbio, Cat# P7630, Beijing, China). All the cells were cultured in a 37 °C incubator with a 5% CO_2_ atmosphere. For short-term starvation, cells were cultured in a DMEM medium (Gibco, Cat#11966025) without glucose supplemented with 0.5 g/L glucose (Sangon Biotech, Cat#A501991, Shanghai, China) and 1% FBS for 24 h before drug treatment. The control medium consists of DMEM with 1 g/L glucose (Gibco, Cat#11965092) and 10% FBS. The etoposide (Cat#S1225), Camptothecin (Cat#S1288), adriamycin (Cat#E2516), NU7441 (Cat#S2638), and RI-1 (Cat#S8077) were purchased from Selleck (Houston, TX, USA).

### 4.2. Antibodies

The antibodies against γH2AX (Cell Signaling Technology, Cat#80312S, Danvers, MA, USA), β-Tubulin (abcepta, Cat# AM1031A, San Diego, CA, USA), Caspase 9 (Proteintech, Cat#10380-1-AP, Rosemont, IL, USA), 53BP1 (Santa Cruz Biotechnology, Cat#SC-22760, Dalas, TX, USA), GAPDH (ABclonal, Cat#AC033, Woburn, MA, USA), Cyclin D1 (ABclonal, Cat#A19038), CDK2 (ABclonal, Cat#A0094), Flag (Proteintech, Cat#20543-1-AP, Rosemont, IL, USA), Top2α (ABclonal, Cat#A16440, Woburn, MA, USA), Top2β (Proteintech, Cat#20549-1-AP), HRP Goat Anti-Rabbit IgG (ABclonal, Cat#AS014, Woburn, MA, USA), HRP Goat Anti-Mouse IgG (ABclonal, Cat#AS003), Alexa Flour^TM^ 488 donkey anti-mouse IgG (H+L) (Thermo Fisher Scientific, Cat#A21202, Waltham, MA, USA), and Alexa Flour^TM^ 594 donkey anti-rabbit IgG (H+L) (Thermo Fisher Scientific, Cat#A21207) were purchased commercially.

### 4.3. Western Blot

The cells were washed with PBS three times and lysed in RIPA buffer. The proteins were separated using 10% SDS-PAGE and transferred onto PVDF membranes (Roche, Cat#52130500, Basel, Switzerland). In addition, after blocking in PBS with 5% skim milk for 1.5 h, the membranes were incubated with the corresponding primary antibodies overnight at 4 °C. After washing the membrane three times for 5 min each time with PBST, incubate the secondary antibody for 1 h at room temperature, and then wash the membrane three times with PBST. The chemiluminescence solution was prepared according to the instructions and added to the PVDF membrane in drops. The images were scanned by the Tanon 4500 Imaging System (Tanon, Shanghai, China) and quantified with ImageJ (National Institutes of Health).

### 4.4. Cell Viability Assay

The cell viability was measured by Cell Counting Kit-8 (CCK-8) from APExBIO (Cat#K1018, Houston, TX, USA). In addition, cells were seeded onto a 96-well plate in 100 μL control medium or short-term starvation medium for 24 h in each well. After treatment with the indicated concentrations of drugs, add 10 μL CCK-8 solution to each well of the plate. The plate was incubated for 1.5 h at 37 ℃ and measure the absorbance at 450 nm using a microplate reader (TECAN infinite F200 PRO).

### 4.5. siRNA Sequences and Transfection

For knockdown of GAPDH, HeLa cells were transfected with scramble siRNA and GAPDH siRNA using ExFect Transfection Reagent (Vazyme, Cat#T101-01, Nanjing, China) for 48 h. The sequences of siRNAs targeting GAPDH are as follows: Scramble siRNA, forward-acuccccuugcucauguauactt, reverse-guacaugagcaaggggagutt; GAPDH-siRNA, forward- ugaccucaacuacaugguutt, reverse-aaccauguaguugaggucatt. All the vectors used in this article are prepared from the SPARKeasy Endofree Midi Plasmid Kit (SparkJade, Cat#AD0105, Jinan, China).

### 4.6. Immunofluorescence

The cells were grown on coverlips in 12-well plates, fixed with 4% paraformaldehyde for 10 min, permeabilized with 0.1% Triton X-100 (Solarbio, Cat#T8200, Beijing, China) for 10 min, and blocked with 3% BSA (SunShineBio Cat#B0012-100, Shanghai, China) at room temperature for 1 h. The cells were immunostained with primary antibodies against various proteins overnight at 4 ℃. Next, the cells were washed with PBS three times and then stained with the Alexa Fluor 594 or Alexa Fluor 488 conjugated secondary antibodies at room temperature for 2 h. After washing, the cells were stained with DAPI (Bioworld, Cat#AC15221, Nanjing, China) at 37 ℃ for 10 min. Further, after being washed three times in PBS, the coverslips were mounted using an antifading mounting medium (Solarbio, Cat#S2100, Beijing, China). The cells were visualized by a fluorescence microscope (Nikon, 80I 10-1500X), and the images were captured with a charge-coupled device camera.

### 4.7. Apoptosis Assay

The HeLa cells were transfected with scramble siRNA and GAPDH siRNA for 48 h, treated with ETO for 4 h, and then collected and stained with both Annexin V and PE using an Annexin V-PE Apoptosis Detection Kit (KeyGEN BioTECH, Cat#KGA1011, Nanjing, China) according to the manufacturer’s instructions. The apoptosis was analyzed by flow cytometry using the BD FACSverse.

### 4.8. Comet Assay

The cells were harvested at various time points post-ETO and processed for the comet assay using a DNA Damage Detection Kit (SCGE) (KeyGEN BioTECH, Cat# KGA240) according to the manufacturer’s protocol. In addition, cells were photographed under a Zeiss Axiovert 200 M microscope, and comet tail analysis was computed by ImageJ using the OpenComet plugin.

### 4.9. HR and NHEJ Assay

The HR and NHEJ efficiencies were measured using DR-GFP U2OS cells and EJ5-GFP U2OS cells, respectively. The cells were transfected with scramble siRNA and GAPDH siRNA for 12 h, then transfected with the pCAGGS-I-SceI plasmid. Further, the cells were harvested for GFP expression detection by flow cytometry (BD Biosciences). For each analysis, 10,000 cells were processed, and experiments were repeated three independent times.

### 4.10. Statistical Analysis

The statistical analyses were performed with a two-tailed, unpaired Student’s *t*-test. All data shown represent the results obtained from 3 independent experiments with SEM (mean ± SD). ns, not significant, * *p* < 0.05, ** *p* < 0.01, *** *p* < 0.001.

## Data Availability

The data presented in this study are available on request from the corresponding author.

## Supplementary Materials

The following supporting information can be downloaded at https://www.mdpi.com/article/10.3390/ijms24032498/s1.
