# Supplementary material for: Short-Term Starvation Weakens the Efficacy of Cell Cycle Specific Chemotherapy Drugs through G1 Arrest
Source: Int J Mol Sci. 2023 Jan 28;24(3):2498. doi: 10.3390/ijms24032498 (PMC9917170; doi:10.3390/ijms24032498)
Supplement: Supplementary file 1 [file ijms-24-02498-s001.zip › ijms-2086319-supplementary.pdf]

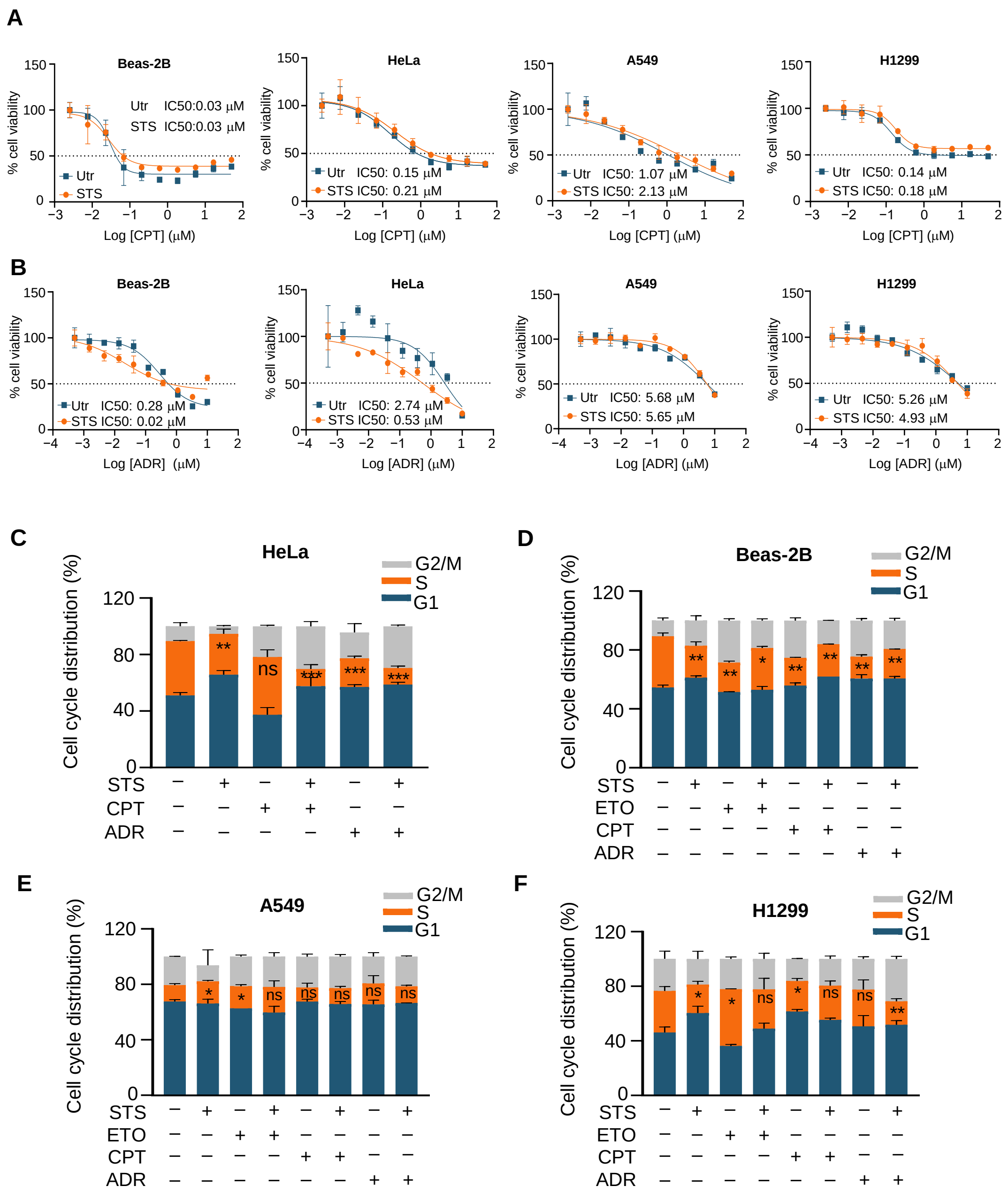

Figure S1. Dose-response curves and cell cycle quantifications of four different cell lines.

(A-B) Dose-response curves with IC50 values of indicate cell lines pre-starved or left untreated for 24 h were treated with the indicated concentration of CPT or ADR for 48 h.

(C-F) Cell cycle quantification of indicate cell lines pre-starved or left untreated for 24 h were treated with the ETO (20 μM, 2 h), ADR (20 μM, 2 h) and CPT (50 μM, 2 h). Data represented as mean ± SD of at least three independent experiments. P values are from student's t-tests. **P*<0.05; ***P*<0.01; ****P*<0.001 and ns: not significant.

A

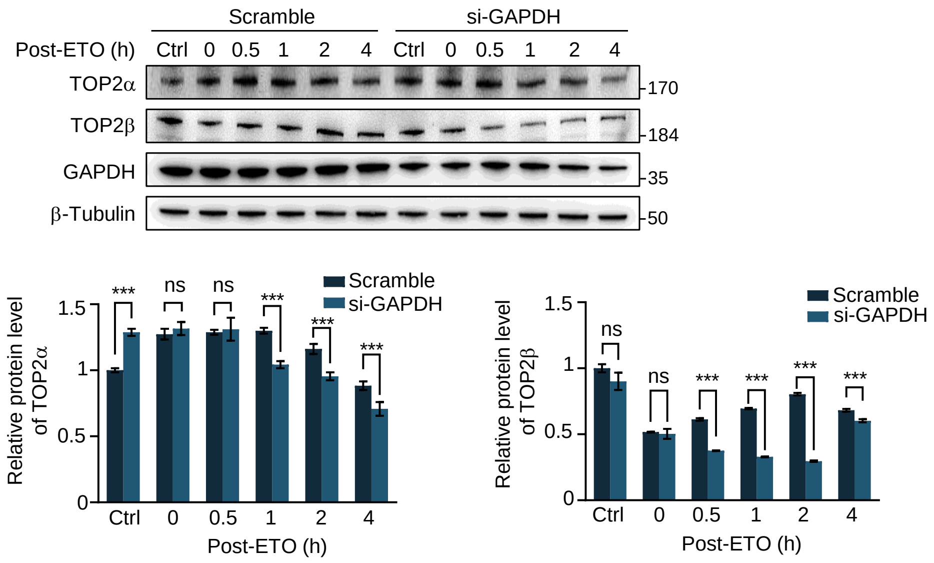

Figure S2. Protein abundances in scramble and GAPDH knockdown HeLa cells. Immunoblot (Upper) and quantification (lower) of indicated proteins in control or GAPDH knockdown HeLa cells with ETO treatment (20 μM, 2 h) for different recovery times. Data represented as mean ± SD of at least three independent experiments. P values are from student's t-tests. ****P*<0.001 and ns: not significant.
